# Motor Imagery for Severely Motor-Impaired Patients: Evidence for Brain-Computer Interfacing as Superior Control Solution

**DOI:** 10.1371/journal.pone.0104854

**Published:** 2014-08-27

**Authors:** Johannes Höhne, Elisa Holz, Pit Staiger-Sälzer, Klaus-Robert Müller, Andrea Kübler, Michael Tangermann

**Affiliations:** 1 Neurotechnology group, Berlin Institute of Technology, Berlin, Germany; 2 Department of Psychology I, University of Würzburg, Würzburg, Germany; 3 Beratungsstelle für Unterstützte Kommunikation (BUK), Diakonie Bad Kreuznach, Bad Kreuznach, Germany; 4 Machine Learning Laboratory, Berlin Institute of Technology, Berlin, Germany; 5 Bernstein Center for Computational Neuroscience, Berlin, Germany; 6 Department of Brain and Cognitive Engineering, Korea University, Anam-dong, Seongbuk-gu, Seoul, Korea; 7 BrainLinks-BrainTools Excellence Cluster, University of Freiburg, Freiburg, Germany; ARC Centre of Excellence in Cognition and its Disorders (CCD), Australia

## Abstract

Brain-Computer Interfaces (BCIs) strive to decode brain signals into control commands for severely handicapped people with no means of muscular control. These potential users of noninvasive BCIs display a large range of physical and mental conditions. Prior studies have shown the general applicability of BCI with patients, with the conflict of either using many training sessions or studying only moderately restricted patients. We present a BCI system designed to establish external control for severely motor-impaired patients within a very short time. Within only six experimental sessions, three out of four patients were able to gain significant control over the BCI, which was based on motor imagery or attempted execution. For the most affected patient, we found evidence that the BCI could outperform the best assistive technology (AT) of the patient in terms of control accuracy, reaction time and information transfer rate. We credit this success to the applied user-centered design approach and to a highly flexible technical setup. State-of-the art machine learning methods allowed the exploitation and combination of multiple relevant features contained in the EEG, which rapidly enabled the patients to gain substantial BCI control. Thus, we could show the feasibility of a flexible and tailorable BCI application in severely disabled users. This can be considered a significant success for two reasons: Firstly, the results were obtained within a short period of time, matching the tight clinical requirements. Secondly, the participating patients showed, compared to most other studies, very severe communication deficits. They were dependent on everyday use of AT and two patients were in a locked-in state. For the most affected patient a reliable communication was rarely possible with existing AT.

## Introduction

Aiming to develop communication pathways, which are independent of muscle activity, the research area of Brain-Computer Interfaces (BCIs, [1], [2]) has significantly emerged over the last two decades. BCIs strive to decode brain signals into control commands, such that even severely handicapped people with no means of muscular control are enabled to communicate. Different types of brain signals can be used to control a BCI and a vast amount of studies have demonstrated the proof of concept, showing that healthy users are able to control noninvasive BCIs with a high accuracy and a communication rate of up to 100 bits/min [3]. Translating brain signals into digital control commands, BCI systems can be applied for communication [4], interaction with external devices (e.g. steering a wheelchair) [5], rehabilitation [6] or mental state monitoring [7], [8]. While recent studies also investigated the neuronal underpinnings of BCI control [9], [10], the main objective of BCIs has always been to provide an alternative communication channel for patients that are in the locked-in state [11]–[13].

Brain signals suitable for BCI can be acquired with numerous acquisition technologies, such as electroencephalogram (EEG), magnetoencephalogram (MEG), functional magnetic resonance imaging (fMRI), functional near-infrared spectroscopy (fNIRS) or electrocorticogram (ECOG) in an invasive and non-invasive manner. While these different approaches are reviewed in [1], [2], [14], we focus on non-invasive BCI systems which are based on EEG signals.

Based on experiments with healthy users, various improvements in the experimental design [15], [16], and on the algorithmic side [17]–[21] have recently been presented. In particular, machine learning methods have been developed to improve feature extraction [18] and classification [22]–[26] of neuronal signals, enabling the field to set up an online BCI paradigm for naive healthy users within a single session. Until now, these improvements have mostly been tested on offline data from healthy subjects.

There are different types of BCI paradigms, which can generally be differentiated in (I) self-driven paradigms and (II) stimulus-driven paradigms. Stimulus-driven paradigms evaluate the neuronal response to multiple stimuli which are presented consecutively. The objective of the BCI is to detect to which stimulus the user is attending. Numerous stimulus-driven paradigms were introduced, with stimuli from the visual [3], [27], [28], auditory [29], [30] or tactile [31] domain and they have proven successful in end-users with severe diseases leading to motor impairment [32], [33]. Moreover, several types of neuronal responses (e.g. evoked potentials and steady-state potentials) enable to differentiate between the brain responses of attended and non-attended stimuli. As these paradigms are all relying on the user's perception of those stimuli, patients with sensory impairments may not be able to use such BCI systems [34].

Self-driven BCI paradigms are not relying on the perception of external stimuli, as these systems are based on brain signals which are intentionally produced by the user. Here, “Motor Imagery” (MI) is a widely used paradigm, in which the BCI detects changes of brain patterns (such as sensory motor rhythms), which are associated with the imagination of movements. In a common MI scenario, a computer can be controlled (e.g. moving a cursor on the screen) through either imagination of movements of the left hand/right hand/foot [35] or their attempted execution.

Although the proof-of-concept for noninvasive BCI technology has already been shown more than twenty years ago, patient studies are still very rare. Kübler (2013) [13] recently pointed out that “fewer than 10% of the papers published on brain-computer interfacing deal with individuals presenting motor restrictions, although many authors mention these as the purpose of their research”. Moreover, within patient studies, those patients who were chosen to participate were rarely in need of a BCI, since their residual communication abilities with assisted technology (AT) were higher than the best state-of-the-art BCI could ever provide. Thus, there is a lack of studies with patients who are in a state that allows the BCI to become the best available communication channel. Some examples can be found in [4], [11], [34], [36]–[44], also being reviewed in [12], [45], [46]. However, recent clinical studies have shown that it is even possible to set up BCI systems with patients in the complete locked-in condition. De Massari (2013) [47] introduced the idea of semantic conditioning as a potential alternative paradigm with completely paralyzed patients, and [48] applied a MI paradigm with patients diagnosed as being in the vegetative state. Moreover, patients with disorders of consciousness were trained to use BCI [49], however, no functional communication could be achieved. These studies reveal that it may be possible to obtain significant classification accuracies for those patients, but it has not yet been shown that patients in complete paralysis can “reliably” use a BCI system [50].

Our contribution describes the results of a MI-BCI study with four patients who showed severe brain damage. While all four patients had substantial difficulties with communication, two patients had a communication rate with their individually adapted AT of less than 5 bits/min. This means that for these participants, a BCI has the chance to become their individually best available communication channel, with all the beneficial implications for the Quality-of-Life of these patients [51], [52].

The objective of this study is to show that the application of state-of-the-art machine learning methods allows to set up a MI-BCI system for patients in need of communication solutions within a very small number of sessions. We addressed this issue within a BCI gaming paradigm, which was specifically adapted to the needs of each patient according to user-centered design principles [53]. Both, the BCI system and the feedback application were optimized in an iterative procedure in order to account for the users' individual preferences. For the first time, automatically adapting classifiers, as well as hybrid data processing and classification approaches were applied online with (locked-in) patients. Moreover, a thorough psychological evaluation was done [51].

More precisely, we demonstrate that by following the principle “let the machine learn,” [54], patients gained significant BCI control within six sessions or less.

## Materials and Methods

### 2.1 Patient Participants

The BCI system was tested with four severely disabled users in the information center of assistive technology, Bad Kreuznach, Germany. The patients were diagnosed with different diseases causing hemi- or tetraplegia. All patients were in a generally constant condition with no primary progress in their disease. No cognitive deficits were known. Table 1 summarizes disease- and demographic-related information. All patients had severe communication deficits and were using an AT solution on a daily basis. They had been continuously provided with individually optimized and cutting-edge AT (such as customized switches or eye-trackers) for more than five years. Only patient 3 had previously participated in BCI with MI training in a different study more than ten years ago - without gaining significant control (see patient *KI* in Kübler (2000) [55] and Kübler & Birbaumer (2008) [12]). It should be noted that the patient numbering was ordered with decreasing residual communication abilities. Two of the four patients (patients 3 and 4) were in the locked-in state. Patients in the locked-in state are restricted in their voluntary motor control to such an extent that they are not able to communicate. This definition however makes an exception for one remaining communication channel. For most patients in the locked-in state, eye movements are the last remaining form of muscular control. If no remaining form of voluntary muscular activity is available (including the control of eye gaze, blink or button press), patients are considered to be in the “complete locked-in state”.

**Table 1:** Demographic and disease related data of all patients.

	Patient 1	Patient 2	Patient 3	Patient 4
Age	47	48	45	45
**D**ia**gnosis**	Tetraparesis after pons infarct	Hemiplegia after cerebral bleeding	Infantile cerebral palsy	Tetraparesis after cerebral bleeding
**Artificial Ventilation**	No	No	No	No
**Artificial Nutrition (PEG)**	No	No	No	Yes
**Wheelchair**	Yes	Yes	Yes	Yes
**Residual muscular control**	Eye-movement Speech Residual movement of right hand	Eye-movement Residual movement of left arm, hand and head Mimic	Eye movement (unreliable) Mimic Residual movement of right hand/arm	Eye-movement (highly unreliable) Residual movement of left thumb (depending on physical state)
**Computer input device**	Keyboard PC	Keyboard PC	Joystick/switch with hand letterboard with eye movements	Button press with thumb (yes/no): yes: 1 button press no: 2 button presses
**Use of ICT on a daily basis**	Yes	Yes	Yes	Yes
**Experience with AT since**	2006	1982	1986	2000
**ITR with AT ICT**	>30 bits/min	>30 bits/min	1–5 bits/min	0–2 bits/min
**Experience with MI -BCI**	No	No	Yes	No

Since different disagreeing definitions of the (complete) locked-in state exist, Table 1 also provides the communication rate with AT (measured as Information Transfer Rate (ITR) in bits/min [56]) as an additional measure. Communication rates with AT were empirically estimated by quantifying the time that the users needed to answer yes/no questions or ratings on a visual analog scale (VAS) in the evaluation process of this study. In the following paragraphs, each individual patient and his current physical condition is described in further detail.

#### Patient 1

Amongst all patients enrolled in this study, patient 1 had the least impaired communication ability – being able to speak. Due to a stroke, his pronunciation is slurred, his language is considerably slowed down and needs to be amplified in volume. Although he has limited control over his left hand, he can reliably control his right hand to write, type or steer an electric wheelchair.

#### Patient 2

Although lacking the ability to speak, patient 2 has high residual communication abilities since he can voluntarily control the left hand, left arm and his facial muscles. Thus, he can gesture and also use a standard computer keyboard.

#### Patient 3

Patient 3 is communicating with trained caregivers (partner-scanning) by controlling his eye gaze. He has been trying to use numerous eye-tracking systems, without gaining sufficient control. However, he can control a computer with a slow, weak but reliable control of his right forearm through the press of a button. Being highly motivated to use BCI technology, he already participated in a BCI study more than ten years ago [55], which tested the control via slow cortical potentials (SCP) of the EEG. Unfortunately, he was not able to gain reliable control over the SCP-based BCI system in any session. Due to highly limited means of communication, a functioning BCI system would directly improve the quality of life of patient 3.

#### Patient 4

Having the goal to provide communication solutions for people who can hardly communicate with AT or otherwise, patient 4 represents the ultimate end-user target group for BCI technology. The one exclusively known voluntary muscular control is a rather unreliable movement of his right thumb. He thus uses his thumb to press a button (pinch grip), which reflects the only available communication channel.

When starting the study, he had been in this condition for more than nine years. His communication is very slow and unreliable to the extent, that he is sometimes completely unable to communicate at all for several hours. In principle, he uses the button press in order to communicate an answer upon a question. A single button press would represent a *yes*-answer/agreement, while disagreements are expressed by two consecutive button presses. He shows a high variation within and across days of his attentiveness (he spontaneously falls asleep), of his mood, and of his responsiveness. The median time for a single button press is estimated to be 12 s, but delays of tens of seconds appear frequently (approx. 40%). The variation of responsiveness is the biggest communication hurdle: whenever patient 4 wishes to provide a negative response or disagreement, the second button press might be heavily delayed or not executed. Then the caregiver erroneously assumes an agreement. Given this communication quality and a communication rate at its best of 2 bits/min, patient 4 can be regarded to be close to the complete locked-in condition.

### 2.2 Study Protocol

**Figure 1:**
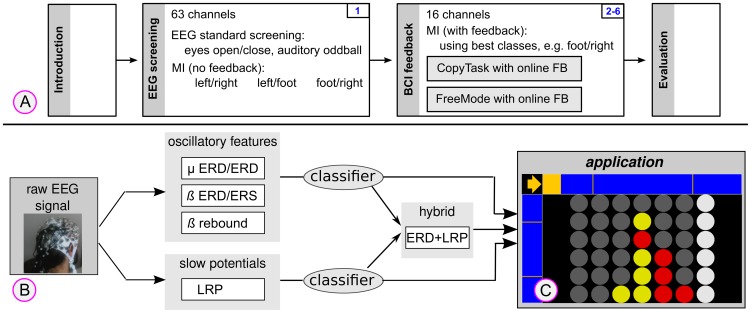
The experimental design is shown in plot (A). Plot (**B**) depicts the architecture of the flexible BCI system which simultaneously considers oscillatory features and slow potentials. Two classifiers are applied and the feedback application is receiving simultaneous output of both classifiers and their weighted combination. A screen shot of the “Connect-4” application in mode *FR* (foot vs. right hand) is plotted in (**C**). In the top-left corner, the cue is presented (an arrow pointing to the right) and based on the BCI output, the yellow bar is either extending rightwards or downwards. The rightmost column is currently selected and visually highlighted.

The study protocol was approved by the Ethical Review Board of the Medical Faculty, University of Tübingen, Germany (case file 398/2011BO2). Written informed consent was obtained from each patient or their legally authorized representative. The study consisted of six EEG sessions per patient. There was not more than one EEG session per day and depending on the patient's condition, the session took 1–3 hours - including preparation time. Additionally, one introductory interview was conducted before the study and two interviews for evaluation were held after the last BCI session. Fig. 1A depicts details of the individual sessions. The psychological evaluation, with respect to the interview and questionnaires, is described in a separate article [51].

In the first EEG session, every patient was screened to explore individual brain patterns and to select the two MI classes (left-hand, right-hand and foot imagery) which resulted in highest and most robust class-discriminability. Moreover, standard auditory oddball ERP recordings and a labeled recording for eye-movements, blinking artifacts and eyes open/closed measurements were performed during this screening session. MI training with feedback was not performed during this first EEG session, but only during the following five BCI sessions.

Each feedback session (2–6) was split in two parts: patients first executed a copy task (CopyTask), afterwards they received full control of the application in the free game mode (FreeMode). Patients 3 and 4 attempted to perform a motor action, while patients 1 and 2 used motor imagery. In each trial, the task was visually cued by an arrow, e.g. pointing rightwards or downwards (for right-hand or foot imagery), see Fig. 1B. During both the CopyTask and the FreeMode, patients received online feedback (see Fig. 1C) of their targeted brain activation. However, in the CopyTask the outcome of a trial did not initiate an action in the game. In the FreeMode, the directional cue was replaced by a question mark and the gaming application was fully controlled by the BCI with two available actions: "select next column" and "place coin". Each action was represented by one MI class. The FreeMode was only started if the patient had reached sufficient control (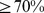) in the CopyTask (leading to less frequent and shorter FreeMode phases for early sessions).

In order to reduce the number of unintended actions in the FreeMode, an action (placement of a coin or selection of the next column) was only performed if a predefined threshold had been exceeded by the BCI classifier. This resulted in "noDecision" trials if the threshold was not exceeded. Consequently no action was elicited for these trials. Introducing "noDecision" trials lead to a decreased fraction of incorrect decisions, yet at the same time to a reduction of communication rate (here: actions per minute and ITR). The ITR values reported throughout this paper were calculated such that all pauses were taken into account [29].

Within the entire study, long durations of trials and inter-trial pauses led to an approximate speed of 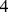 trials/minute. Since one bit can be coded within one trial, the maximum achievable bit rate with this system was about 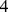 bits/min (with 100% correct trials). Although speeding up the communication rate by shortening the durations of trials and pauses would have been possible, we did not make use of this option in order to minimize the stress level and workload. Moreover, it should be noted that a reliable slow control might be preferable compared to a fast communication solution which is less reliable.

### 2.3 Application

Gaming applications represent a playful way to practice and improve the use of BCI systems, because they may provide long-term and short-term motivation. Moreover, we considered the frustration of erroneous actions in a game to be lower than erroneous selections of letters in a spelling task. Therefore, a computer version of the game “Connect-4” was used within all sessions. “Connect-4” is a strategic game, in which two players take turns in filling a matrix of free slots with coins. The objective of the game is to connect four of one's own coins of the same color vertically, horizontally, or diagonally. The two players are alternately placing their coins in one of the seven columns. The gaming application can be controlled by a 2-class motor imagery BCI, since only two actions are needed to play the game: (1) select the next column, or (2) place the coin in the current column. The software was implemented as a standalone java-application. Fig. 1C shows a screen shot of the application.

### 2.4 EEG acquisition

Two different EEG systems were used within this study, both systems utilized passive gel electrodes. In the screening session, a 63-channel EEG system was used with most electrodes placed in motor-dense areas (cap: EasyCap, amplifier: BrainProducts, 2
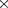
32 channels, 1000 Hz sampling rate). One EOG channel was recorded additionally below the right eye. In sessions 2–6, a 16-channel EEG system was used (cap&amplifier: g.Tec, 1200 Hz sampling rate), while electrodes were placed symmetrically in areas close to the motor cortex. All EEG signals were referenced to the nose. Impedances were kept below 10 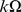, if possible. Data analysis and classification was performed with MATLAB (The MathWorks, Natick, MA, USA) using an inhouse BCI toolbox. For online processing and offline analysis, the EEG data was low-pass filtered to 45 Hz and down-sampled to 100 Hz.

### 2.5 BCI setup

This study focused on patients with severe brain injuries, thus the EEG signals and class-discriminative features were expected to be different to those known for healthy users. For this reason, the BCI was designed such that it could be driven by a wide range of features and their combinations. The incorporation of multiple features of the EEG or from other modalities into the BCI system is called a “hybridBCI” system, which is a rather recent line of research [57]–[59]. Fig. 1B shows the architecture of the BCI system used for this patient study. The BCI simultaneously delivered three control signals to the application. Spectral features (event related desynchronization (ERD) in 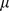, 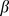, 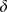 band or 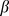 rebound) as well as slower movement-related potentials (i.e. lateralized readiness potential, LRP) were processed and classified. The two classifier outputs and their individually weighted sum were received by the application. The experimenter could then choose (based on a prior offline analysis of the data), which of the three output signals should be used to control the application.

### 2.6 Feature extraction and classification

To extract oscillatory features, signals were band-pass filtered by a Butterworth filter of order 5 in the individually defined spectral band. After visual inspection of the channel-wise ERD, a discriminative time interval was defined to compute optimized spatial filters with the Common Spatial Patterns (CSP) method [60] and to train the classifier, a shrinkage-regularized linear discriminant analysis (LDA) [18]. In analogy to Blankertz and colleagues (2008) [60], offline classification accuracy was estimated using a (standard) cross-validation procedure, where the CSP filters and LDA weights were computed on the training set, and binary accuracy was assessed on the test set.

For the feature extraction of non-oscillatory slow potentials, raw EEG was band-pass filtered with a Butterworth filter (0.2–4 Hz) with a subsequent channel-wise baselining step (the interval of 300 ms duration before trial onset). In analogy to ERP classification [18], the mean amplitude in a manually selected (class-discriminative) time interval was taken from each channel in order to form the feature vector of a trial. A binary classifier (again LDA) was trained based on those features.

Both LDA classifiers were automatically adapted during the CopyTask phase. As described in [22], the pooled covariance matrix and the mean of the features was re-estimated after each trial, using the known labels (adaptation rate of 0.03). This also resulted in an implicit bias correction. In the FreeMode, no adaptation was performed. Besides the internal adaptation, the research team could recalibrate and fine-tune the classifiers between and within sessions. This was important in order to account for unstable features in the EEG data.

## Results

### 3.1 Standard screening

**Figure 2:**
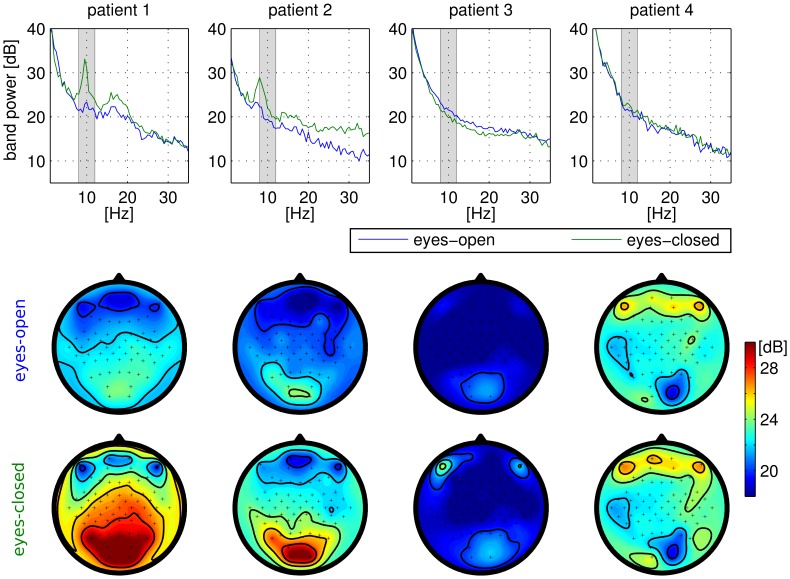
Standard physiological screening of the four patients. The top row shows the spectra at electrode ‘
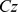
’ in the conditions eyes-open and eyes-closed. The spatial distribution of the channel-wise spectral power in the alpha-band [8–12 Hz] is depicted in the scalp maps of the lower row.

The outcome of the standard screening (session 1) is depicted in Fig. 2. For patients 3 and 4 we found very atypical EEG signatures without any alpha or beta rhythms in the eyes-open and eyes-closed condition. It should be noted that these patients were unable to voluntarily open and close their eyes in response to an instruction/cue. Thus, eye-closure was supported by the caregiver who carefully moved the eyelids by hand.

### 3.2 ERD features and BCI performance

The BCI performance in this study was assessed for the two experimental conditions: during the CopyTask, the labels are known and the BCI performance can easily be evaluated using the fraction of correct trials (called “binary accuracy” in the following). A trial is correct, whenever the accumulated BCI output is pointing to the correct direction at the end of the trial, thus chance level is 50%.

For the FreeMode, labels are unknown, unless the patient is able to report his intention with AT in each trial. Moreover, the number of games which were won against a computer heuristic can also be assessed as a complex and very high-level performance measure for the FreeMode. Playing the game with random control was simulated with the finding that a random player won 10% of the games and 20% of the games ended with a draw. Thus, the computer heuristic would win 70% of the games when playing against a player with random control.

#### Offline analysis

**Figure 3:**
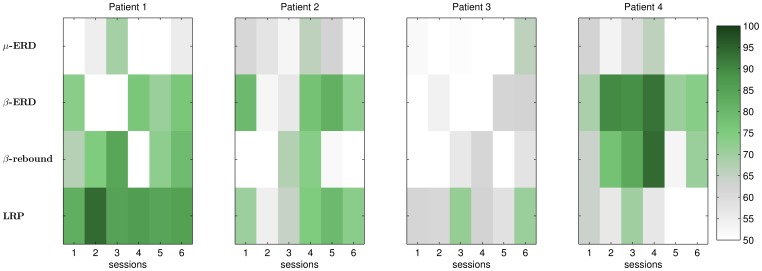
Discriminative power of each feature across sessions, obtained with offine reanalysis of the CopyTask data. Global parameters such as the frequency band and time interval were chosen individually for each patient after manually inspecting the data from all sessions. For each session, the same global parameters were taken – which might be suboptimal. The classification accuracy was then estimated with cross validation using the same parameters for each session. Note that the number of trails was varying across sessions with later sessions featuring less trials. Moreover, a *β* rebound was defined to as a discriminative feature in the *β* band, which was observed more than 500 ms after the end of a trial. As the *β* ERD of patient 4 was heavily delayed, it is also considered as *β* rebound in this analysis. Fig. S2 shows the corresponding spatial distribution of discriminative information as scalp maps.

One interesting question was whether or not class discriminant features are found consistently across sessions. Therefore, Fig. 3 shows the results of an offline analysis of the CopyTask data. For all patients except patient 3, we found at least one discriminative feature (e.g. 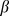 ERD) which was consistently present in all sessions. Patients 3 did not present any reliable feature with discriminative information. Notably, none of the patients featured a consistent ERD component in the 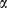 band. However, the spatial distribution of such features was observed to be variable for some patients. Fig. S2 visualizes the spatial distribution of class discriminative information for each patient across all sessions as scalp maps. This finding underlines the necessity of a flexible BCI system like it was used for this study. It should also be noted that the offline accuracy described in Fig. 3 cannot be directly translated into online BCI performance, as the cross-validation procedure was performed for each session separately. The resulting online BCI performance can be lower, if the features changed between sessions [61]. In a scenario of rather stable features across sessions, the online performance can also be higher, as the online classifier was trained with more data (from previous sessions).

#### Online BCI control

**Figure 4:**
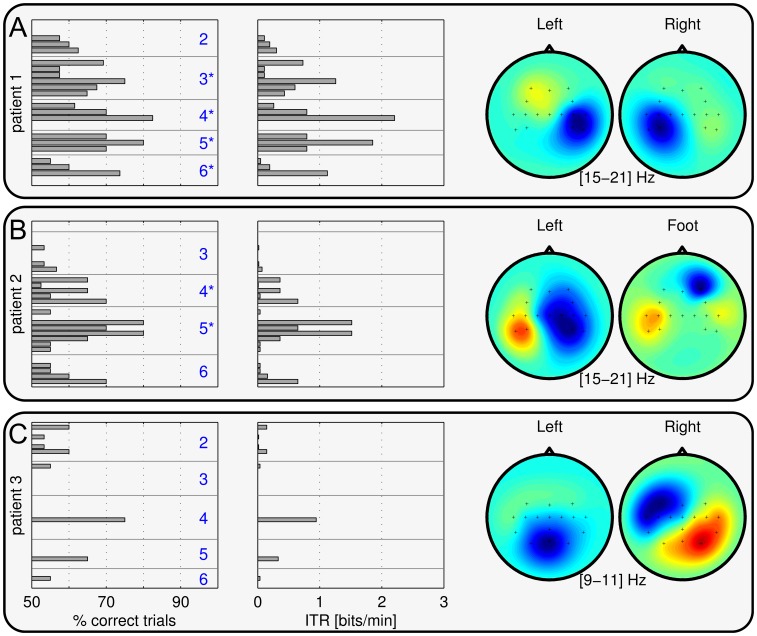
Binary online accuracies (left column) and estimated bit rates (middle column) in the CopyTask for patients 1–3. Each bar represents one block of at least 20 trials. Session numbers are specified in blue color (left column). Session numbers with a * mark sessions with significant online BCI control across all trials (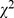 test with *p*<0.05). For patient 2, results for session 3 had to be disregarded due to technical problems. The right column depicts the scalp patterns of the most discriminant spectral features, based on data from all sessions. Results for Patient 4 are shown in Fig. 5.

**Figure 5:**
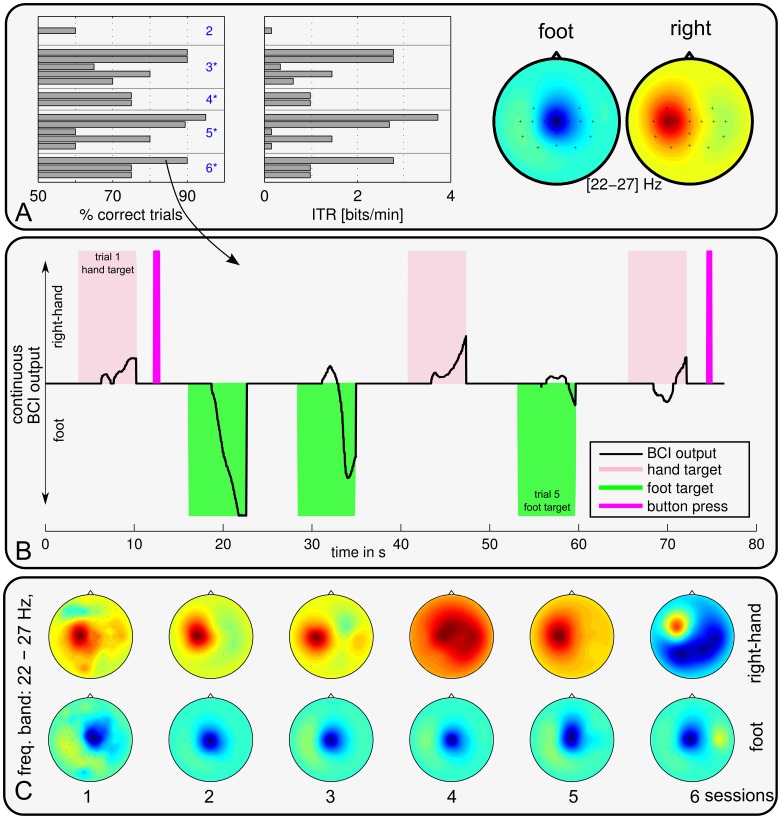
BCI performance and scalp patterns of patient 4. Online binary accuracies, estimated bit rates (left, middle) of the CopyTask, and CSP patterns (right) averaged across all sessions are depicted in the top row (**A**). Each bar represents one block of at least 20 consecutive trials. Middle row (**B**) relates the continuous online BCI output to the residual muscle control (button press) for a representative time segment. Colored areas mark trial periods where the patient was asked to initiate a motor action. The excerpt shown was extracted from session 6, revealing that the BCI can detect the users intention far before a muscle contraction can be initiated. The lower row (**C**) depicts the motor related patterns in the *β* band for each session individually.

Fig. 4 and Fig. 5 show the online performance of the CopyTask for all four patients. All patients except patient 3 could gain significant control over the BCI. Excluding patient 3, we obtained 10/14 sessions with an online binary accuracy being significantly better than chance. Again, one should stress that this was done with a patient population and there were no more than six EEG sessions with each patient, and five of these with BCI feedback. Fig. S3 depicts the online accuracy in the FreeMode, which could only be assessed for patient 1 and 2.

In the following, EEG features and the resulting BCI performance for each of the four patients are discussed separately. Text S1 elaborates on the exact parameterization of the classifiers, which were used in the online study. After previously discussing offline results, we will only discuss online performances in the following.

#### Patient 1

Within the motor imagery study, a beta rebound as well as an LRP were found to be class-discriminant features for left-hand vs. right hand imagery, see Fig. 3. In the online framework, the beta-rebound was used to drive the system in session 4 and all following sessions. The LRP feature was not used, because it was more prone to (eye) artifacts and the patient featured involuntary eye-movements in the directions of the arrow. Although the beta-rebound was found quite consistently, the spatial distribution differed across sessions, see Fig. S2. Therefore, it was required to retrain CSP filters and to use LDA with adaptation. The user was then able to gain significant online control over the BCI, as shown in Fig. 4A. One can also observe that the BCI accuracy increased within sessions, resulting in the most reliable control towards the end of each session. The level of control was not perfect, but sufficient to drive the application in the FreeMode (cp. Fig. S3). Patient 1 played the game Connect-4 five times in total, and he could win three of those games.

#### Patient 2

A beta ERD as well as a LRP were found to be class-discriminant features for left-hand vs. foot imagery, see Fig. 3. Since the beta ERD had a more consistent spatial pattern and was also less susceptible to artifacts, either the beta classifier or the meta classifier (beta + LRP) was used in the online BCI framework. However, although the ERD feature in the beta-band was found in almost every session, one could observe a high variation in class discrimination, spatial patterns as well as in BCI performance across and within sessions (see Fig. 4B and Fig. S2). Due to the adaptive methods mentioned above, patient 2 was nevertheless able to control the game in the FreeMode at the end of session 4 and all following sessions (Fig. S3). In total, he played four games in the FreeMode (winning two of them).

#### Patient 3

In analogy to a previous study [55], reliable class discriminant features could not be found in the EEG data of patient 3 (cp. Fig. 3). He was thus not able to control the BCI system, as shown in the CopyTask performance in Fig. 4C. For the online framework, either the meta classifier or the LRP classifier were applied. None of them performed reliably above chance level. Recall, that this user displayed very atypical EEG spectra at rest (Fig. 2): during the eyes-open and eyes-closed conditions, no alpha or beta peaks were present. Due to the lack of BCI control, patient 3 did not officially enter the FreeMode (see study protocol). However, although featuring insufficient BCI control, patient 3 insisted in attempting to play the BCI game in the FreeMode (“for the fun of it”). He could neither gain control, nor was the resulting data analyzed in the present evaluation.

#### Patient 4

A highly discriminative 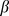 ERD component was present during each session of patient 4 (cp. Fig. 3). His motor-related EEG patterns exhibited typical spatial distributions (see Fig. 5A). This finding is even more surprising, since patient 4 revealed very atypically EEG signatures in the resting state – stereotypical brain rhythms such as 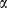 and 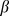 were absent (cf. Fig. 2).

Despite his physical condition, patient 4 achieved the best BCI control amongst the four patients. Fig. 5A shows the online binary performance, revealing that he gained highly accurate online control (up to 90% binary accuracy) over the BCI system within the third EEG session (which was the second session with BCI feedback), and all following sessions. Even when pooling across all six sessions, his BCI control was highly significant (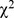 test with 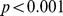). He exhibited very typical EEG activity during the right-hand and foot tasks of attempted motor execution, even though he had been unable to move his feet for more than nine years.

For this patient we could directly compare the communication rate of the BCI to his residual communication abilities with AT, by asking him to execute a button-press as soon as the corresponding cue appeared: we found, that the BCI-controlled feedback became discriminant after 1–3 seconds, while the button-press had a delay of 5–20 seconds — and sometimes the muscle contraction did not occur at all. As an example for this unbalanced communication behavior, a representative time window of 77 s was extracted for Fig. 5B. The interval contains six trials (three hand and three foot trials). The patient was requested to perform a button press in hand movement trial (marked in light magenta), but not during foot trials (marked in green). The BCI output and successful button presses are visualized. Patient 4 could only initiate a thumb muscle contraction successfully in two of the three trials. Moreover, any resulting button presses during this test were considerably delayed and occurred after the trial period of 7 s. The BCI, however, indicated the correct decisions at the end of each trial and even earlier in most cases. For the foot class, no motor action (i.e. muscle movement) was available; nevertheless the BCI could reliably detect the intention of a foot movement. Thus, to the best knowledge of the authors, this is the first quantitative report that shows that a BCI can uncover a patient's intention quicker and more reliable than the best available non-BCI AT.

Due to fatigue, temporal constraints and severe attention deficits, patient 4 entered the FreeMode only twice (sessions 4 and 6). In these two FreeMode sessions, he was not able to stay focused for more than 70 trials. As Table 1 reveals, he had the most severe deficits in communication. In practice, this means that he was mostly unable to communicate his intended action in the FreeMode. As a result, labels of the trials were not available and a data-driven evaluation of his BCI control in the FreeMode was impossible.

## Discussion

Four end-users with severe motor restrictions, who heavily depended on AT for communication and interaction in their daily life, agreed to participate in this study. Two of them were impaired in their communication ability to an extent, that no available AT would enable a reliable and – given their physical state – high speed solution. For these two specific patients, a BCI-based solution for control and communication would indeed introduce a novel communication quality. The BCI could enable independent communication and thus represent an added value compared to the AT presently used.

During the course of six BCI sessions, we found that three out of the four subjects could gain significant BCI control using motor imagery. For the most severely impaired patient (patient 4), we found evidence that the BCI outperformed his existing communication solution with AT in terms of accuracy and information transfer – being discussed in a following section.

The chosen end-user environment posed severe limitations in terms of user availability, their concentration span and the communication quality with their standard AT. We responded to these challenges with a flexible BCI framework, enabling us to tailor three major components of the study to the individual needs of the patient: (1) details of the experimental MI paradigm, (2) the form of data processing and type of exploited brain signals, and (3) the software application, which the user interacted with. Many of the internal modules of the BCI system could flexibly be exchanged and such changes remained invisible to the patients. The result was an "out of the box" BCI system, which was adapting itself to the features and needs of each user. Thus, our BCI system was generic and adaptive to meet the extensive requirements of such a pragmatic patient study.

### 4.1 Reducing the number of sessions using machine learning

With our study we could show, that end-users are able to gain significant online BCI control within six sessions or less. Compared to other end-user studies [38] this is a very low number of sessions. Such a purposeful study design was enabled by the intense combined efforts of those users and the team, consisting of caregivers, psychologists, programmers and data analysts. We thereby followed the principles of user-centered design which implies an iterative process between developers and end-users of a product (see [53]). Thus, we used a setup which was flexible enough to adapt to the user's abilities and needs (e.g. choice of MI-classes, temporal constraints or the type of EEG feature such as ERD, 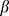-rebound or LRP). Therefore, the system was designed to accommodate a wide variety of end users. Far from downplaying those individual contributions, the positive effect of advanced machine learning (ML) methods, such as hybrid classifiers with adaptation, should be mentioned. While motor-related BCI tasks are known to require a larger number of user training sessions compared to more salient ERP paradigms [38], [44], [62], we managed to apply our BCI system successfully within less than 6 sessions in three cases. While for one participant, no BCI control could be established, the remaining three participants gained sufficient online control to play the game relatively early on. (Patient 1: control from session three onwards, Patient 4: control from session four, and Patient 2: control from session five on.) The reduced time effort before BCI control was established represents a crucial step for bringing BCIs closer to clinical application for users in-need. In a comparable study with locked-in patients by Kübler et al. (2005) [38], machine learning methods were not applied. Reliable performance was achieved only after a substantial number of sessions.

### 4.2 Patient 4

The case of patient 4 deserves special attention. While displaying severely impaired communication abilities, his level of BCI control was en par with very good unimpaired BCI users performing motor imagery.

This is presumably the most exciting finding of the current study, given that practically the full spectrum of AT solutions had been tested for this patient over the past nine years by AT experts. It should be noted that also ERP based paradigms were tested with patient 4 after the presented MI study. Discriminant ERP components could neither be found for a visual multi-class paradigm (MatrixSpeller [63]) nor for an auditory ERP paradigm [64]. The only applicable AT solution (the pinch-grip button press) provided a limited one-class signal with low accuracy and high temporal variability. Nevertheless, the BCI-controlled signal was relatively robust (with up to 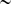
90% accuracy) and available after 7 seconds at the latest.

Evaluating the speed and accuracy of his BCI control, we found evidence that the BCI could outperform his existing communication solution with AT in terms of accuracy and information transfer: during the online CopyTask, patient 4 accomplished commands which were presented visually through the software interface. Interestingly, he used *the same* (attempted) motor command for the right hand BCI class (i.e. the thumb movement) as for a real button press. Thus, a comparison of temporal dynamics and reliability of his BCI-responses with his button-press responses revealed interesting insights, as shown in Fig. 5B.

Contrary to the CopyTask mode, we could not show that patient 4 gained reliable control during the FreeMode. Even though the exact reason for this problem could not be clarified given the limited amount of data available for patient 4, the following – potentially accumulating – causes can be speculated: (1) identification problem, (2) attention problems and fatigue, (3) mental workload (4) self-initiation of actions. Text S2 discusses all mentioned aspects in further detail.

## Conclusions

We could show that patients with severe motor impairments – even patients that are locked-in and almost completely locked-in – were able to gain significant control a noninvasive BCI by motor imagery. While applying state-of-the-art machine learning methods, this control was achieved within six or less sessions. The BCI was then used to operate a gaming application.

These findings are encouraging, since providing communication channels for patients in-need resembles the major goal of the interdisciplinary research field of BCI. Moreover, our study describes one patient (patient 4), whose communication abilities with existing AT were on the same performance level (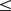 2 bits/min) than his BCI control. In a controlled CopyTask framework, we found evidence that the BCI could even outperform his existing AT solution in terms of accuracy, reaction times and information transfer. Thus, we showed for this patient that neuronal pattern detection of an attempted motor execution can indeed be faster than the muscular output. Future studies may evaluate the BCI control in follow-up sessions, also testing spelling applications. Moreover, broader patient groups will considered in order to further explore and evaluate the clinical usage of BCI.

## Supporting Information

Figure S1
**Description of the different classifiers used within for online BCI.** Across and within sessions, the classifier was retrained on varying subsets of the data and different features. One classifier is described by the set of two neighboring lines (back and blue), a cross in magenta and the number in red. The black lines mark the chosen frequency band, the blue lines mark the time interval used to train and apply the classifier. The cross marks the accuracy of the classifier, estimated with cross-validation on training data. The number in red specifies the number of trails which were used to train the classifier. Note that beginning with the 6th session, the trial length for patient 1 was shortened to 3.5 seconds - resulting in a classification interval after the end of the trail (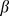 rebound). For all other patients the trial length was 5–7 seconds.(TIF)

Figure S2 **Class discriminant information for each patient across sessions.** For each session, the spatial pattern of the most (left) and second-most (middle) discriminant CSP filter is depicted. Therefore, the same frequency band as well as the same time intervals were chosen for one subject and all sessions. The same parameters were used to generate Figure 3. The right scalpplot visualizes class discrimination of the LRP feature. The classification accuracy of the spectral (CSP-based) classifier and the LRP classifier is printed next the scalpplots. This classification accuracy is estimated with a 5 fold cross validation and gives a quantification of how separable the data was in the corresponding session. In the online scenario, a different classifier was used which was trained on more trails from preceding sessions. Note that the sign of the scalpmaps is arbitrary, thus red and blue (as well as their corresponding graduations) are exchangeable. Note that two colorbars (for CSP patterns and LRP discrimination) are given in the legend. The abbreviation “ssAUC” stands for a signed and scaled modification of the area under the curve (AUC).
(TIF)

Figure S3 **BCI performance in the FreeMode. Patient 1 and patient 2 could communicate their intentions with AT.** Their comments were used as labels for trials in the FreeMode. Note that the scaling of the bitrate is on the right axis. The patients did not enter the FreeMode in session 3 and session 4.
(TIF)

Text S1 **Session to session transfer.**
(TXT)

Text S2 **Discussion of the performance of patient 4 in the FreeMode.**
(TXT)
