# Supplementary figures and images for: Motor Imagery for Severely Motor-Impaired Patients: Evidence for Brain-Computer Interfacing as Superior Control Solution
Source: PLoS One. 2014 Aug 27;9(8):e104854. doi: 10.1371/journal.pone.0104854 (PMC4146550; doi:10.1371/journal.pone.0104854)

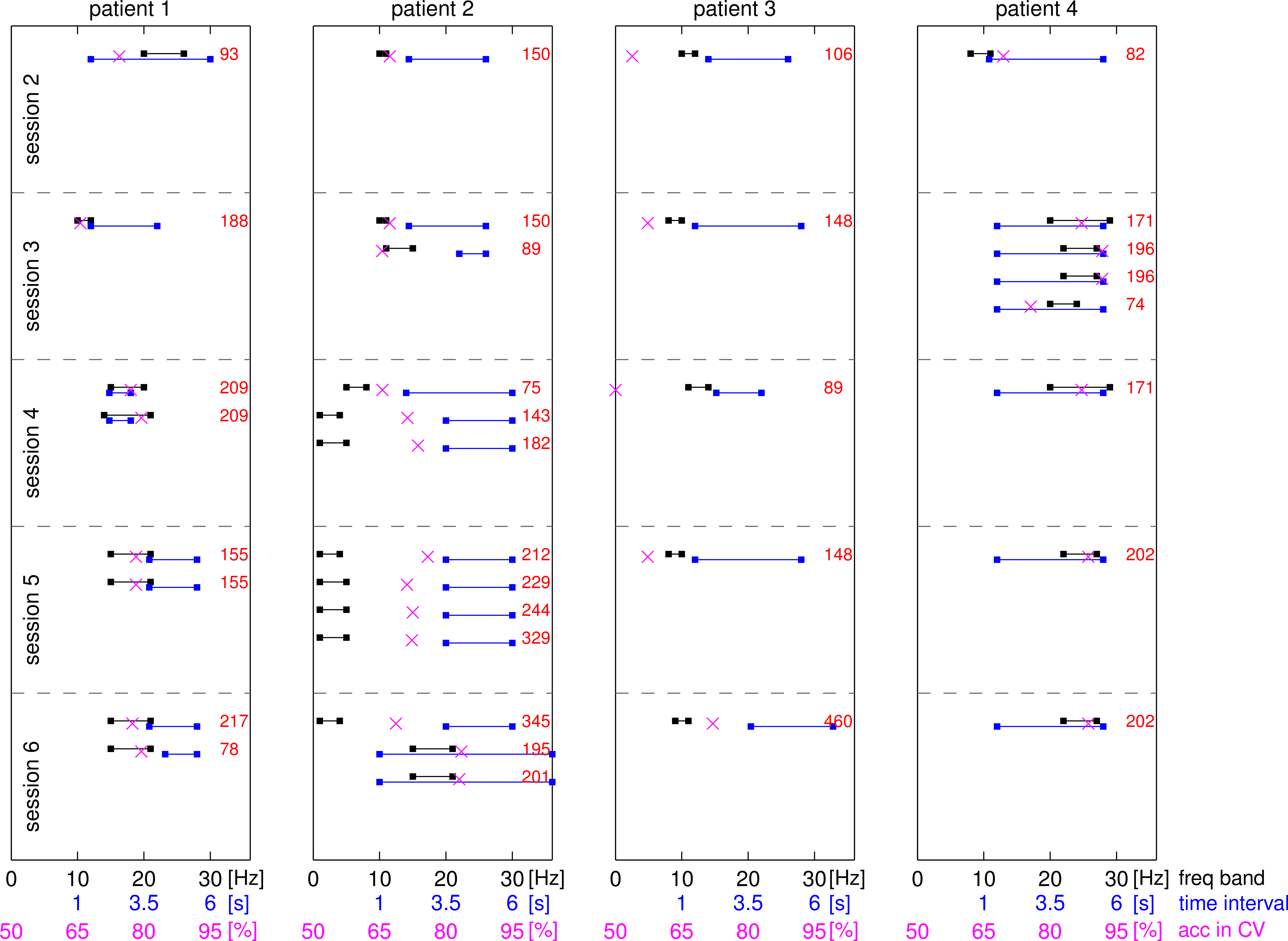

Supplement: Figure S1 — Description of the different classifiers used within for online BCI. Across and within sessions, the classifier was retrained on varying subsets of the data and different features. One classifier is described by the set of two neighboring lines (back and blue), a cross in magenta and the number in red. The black lines mark the chosen frequency band, the blue lines mark the time interval used to train and apply the classifier. The cross marks the accuracy of the classifier, estimated with cross-validation on training data. The number in red specifies the number of trails which were used to train the classifier. Note that beginning with the 6th session, the trial length for patient 1 was shortened to 3.5 seconds - resulting in a classification interval after the end of the trail ( rebound). For all other patients the trial length was 5–7 seconds. (TIF) [file pone.0104854.s001.tif]

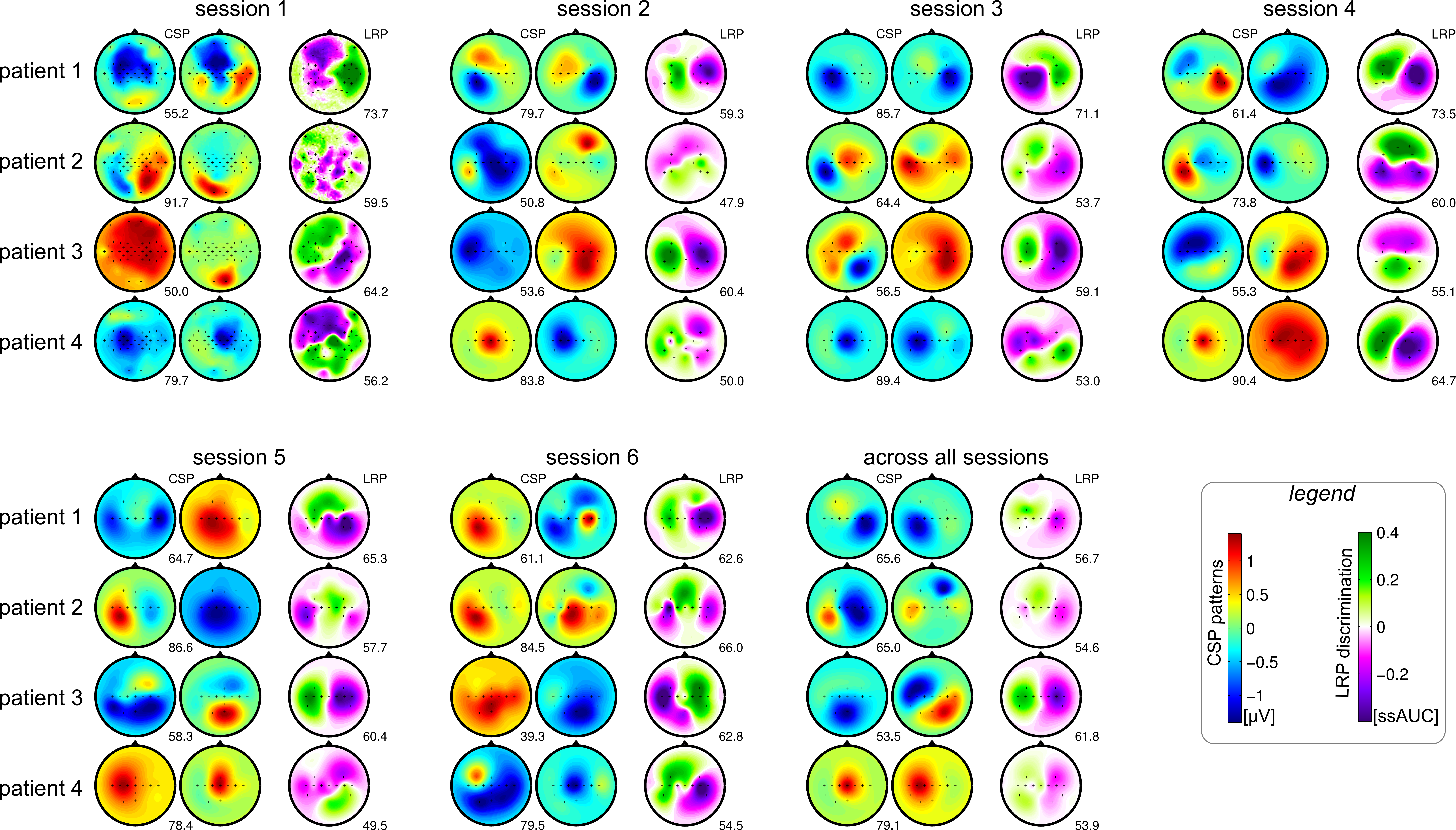

Supplement: Figure S2 — Class discriminant information for each patient across sessions. For each session, the spatial pattern of the most (left) and second-most (middle) discriminant CSP filter is depicted. Therefore, the same frequency band as well as the same time intervals were chosen for one subject and all sessions. The same parameters were used to generate Figure 3. The right scalpplot visualizes class discrimination of the LRP feature. The classification accuracy of the spectral (CSP-based) classifier and the LRP classifier is printed next the scalpplots. This classification accuracy is estimated with a 5 fold cross validation and gives a quantification of how separable the data was in the corresponding session. In the online scenario, a different classifier was used which was trained on more trails from preceding sessions. Note that the sign of the scalpmaps is arbitrary, thus red and blue (as well as their corresponding graduations) are exchangeable. Note that two colorbars (for CSP patterns and LRP discrimination) are given in the legend. The abbreviation “ssAUC” stands for a signed and scaled modification of the area under the curve (AUC). (TIF) [file pone.0104854.s002.tif]

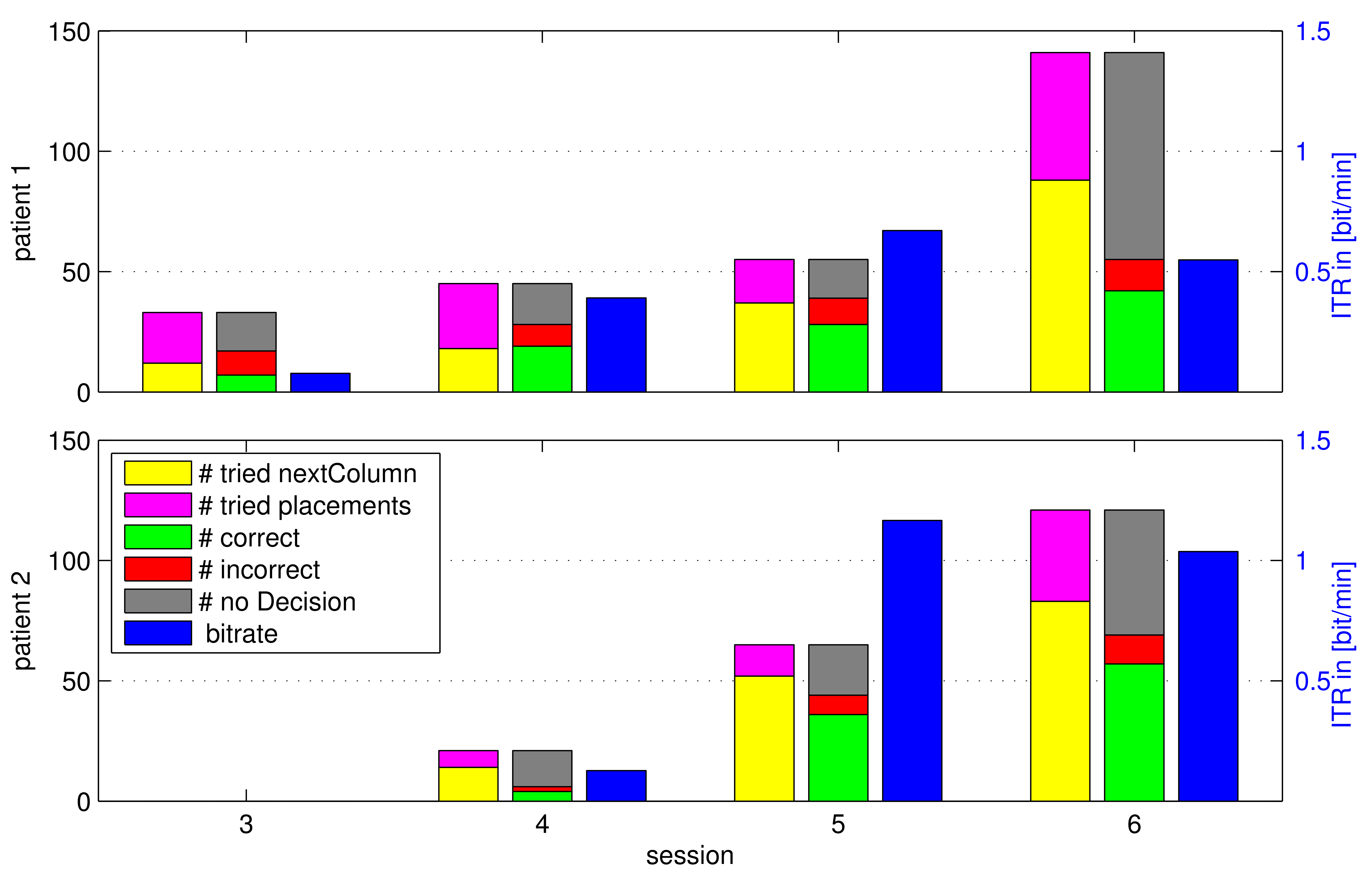

Supplement: Figure S3 — BCI performance in the FreeMode. Patient 1 and patient 2 could communicate their intentions with AT. Their comments were used as labels for trials in the FreeMode. Note that the scaling of the bitrate is on the right axis. The patients did not enter the FreeMode in session 3 and session 4. (TIF) [file pone.0104854.s003.tif]
